# Metabolic syndrome and cardiovascular morbidity in patients with congenital adrenal hyperplasia

**DOI:** 10.3389/fendo.2022.934675

**Published:** 2022-08-01

**Authors:** Mattia Barbot, Pierluigi Mazzeo, Martina Lazzara, Filippo Ceccato, Carla Scaroni

**Affiliations:** Endocrinology Unit, Department of Medicine-DIMED, University-Hospital of Padova, Padova, Italy

**Keywords:** congenital adrenal hyperplasia (CAH)–21-alpha hydroxylase deficiency, cardiovascular risk, metabolic syndrome, diabetes mellitus, obesity, glucocorticoid therapy

## Abstract

Since the introduction of glucocorticoid (GC) replacement therapy, congenital adrenal hyperplasia (CAH) is no longer a fatal disease. The development of neonatal screening programs and the amelioration of GC treatment strategies have improved significantly life expectancy in CAH patients. Thanks to these achievements, CAH patients are now in their adulthood, but an increased incidence of cardiovascular risk factors has been reported compared to general population in this stage of life. The aim of CAH treatment is to both prevent adrenal insufficiency and suppress androgen excess; in this delicate balance, under- as well as overtreatment might be equally harmful to long-term cardiovascular health. This work examines the prevalence of metabolic features and cardiovascular events, their correlation with hormone levels and GC replacement regimen in CAH patients and focuses on precocious markers to early detect patients at higher risk and new potential treatment approaches.

## Introduction

**Figure 1:**
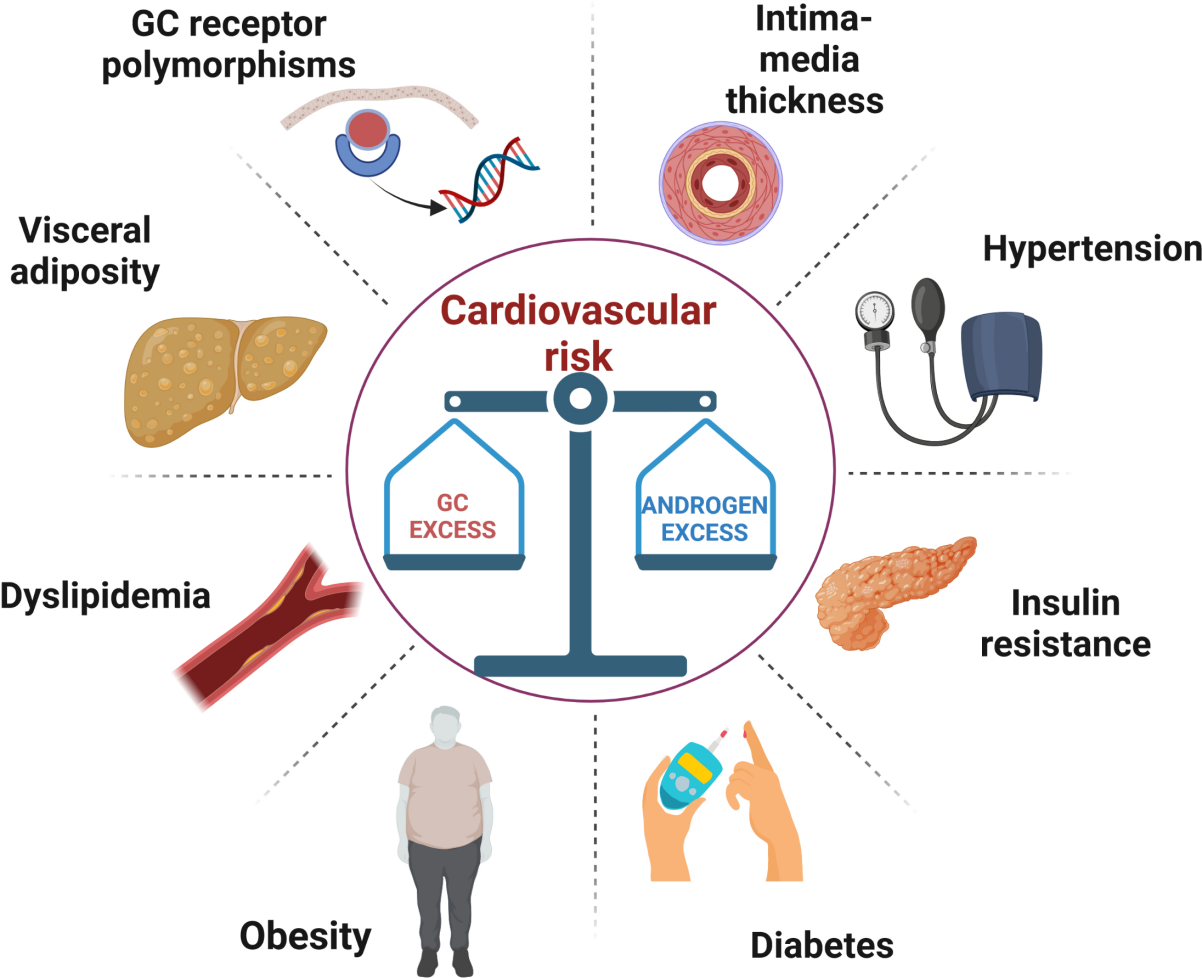
A schematic illustration of the metabolic and vascular complications caused by the difficult balance between glucocorticoid overtreatment and androgen excess in congenital adrenal hyperplasia. GC, glucocorticoid.

Congenital adrenal hyperplasia (CAH) due to 21-hydroxylase deficiency (21-OHD) is an autosomal recessive disorder characterized by impaired cortisol secretion and androgen excess (1). 21-OHD is by far the commonest cause of CAH, accounting for around 95% of cases. On the basis of residual enzymatic activity, 21-OHD is classified into classic (CCAH) and non-classic CAH (NCAH), with the latter usually diagnosed later in life. Depending on the presence or absence of aldosterone deficiency, CCAH is classified into salt wasting (SW) and simple virilising (SV) forms, respectively. The mainstay of CAH treatment, especially for CCAH, is glucocorticoid (GC) and mineralocorticoid (MC) replacement to avoid adrenal crisis and manage androgen excess. The prevention of long-term metabolic and cardiovascular (CV) complications is based on the delicate balance between these two therapeutic objectives (2). In fact, both under- and over-treatment can be equally detrimental for cardio-metabolic health, **Figure 1**. In this short review, we summarized available data on cardiometabolic health in CAH patients, examined the prevalence of predisposing factors and the mechanisms that promote their development.

## Obesity

Obesity is an important independent CV risk factor and is the most frequent component of metabolic syndrome in both children and adults with CAH. Several studies reported a high incidence of obesity, ranging between 30 and 40%, in patients with either classic and non-classic CAH (3–8). Underlying causes include intrinsic susceptibility to adipose tissue accumulation, adipokines imbalance and high leptin levels (4, 9). Notably, in individuals with CCAH, the tendency to precocious adiposity rebound is present in childhood and may persist in adulthood (10, 11). Additionally, CAH patients are prone to increases in visceral adipose tissue, a clear-cut risk factor for CV diseases. Recently, increased visceral adipose tissue (VAT)/subcutaneous adipose tissue (SAT) ratios were found in adolescents with CAH using computed tomography scan (12), although controversial data were found findings by other studies using dual x-ray absorptiometry (13–15).

A possible explanation for the higher adiposity may be due to lower catecholamine levels in CAH, with a consequent reduced lipolysis, especially in the classic form (4). Indeed, in a large cohort of 203 adults patients, higher BMI’s were found in females with CCAH compared to those with NCCAH (3).

Moreover, it was hypothesized that increased BMI could be related to both type and dose of GC therapy, with dexamethasone displaying the worst metabolic profile (16). Despite this, current Guidelines do not recommend one singular form of GC over another in adult patients with CAH (2). Interestingly, in the study by Falhammar et al. which included adult patients, most had isolated obesity in the absence of other CV risk factor (17). The influence of genotype on metabolic imbalance was also studied, but no correlation was established between the degree of enzymatic deficiency and obesity (18). In such a complex scenario, lifestyle and family history should always be considered as potential contributors to obesity; Torky et al. indeed showed a significant association between the presence of obesity in adult CAH patients and maternal obesity (19). Since obesity represents the main risk factor for the development of other metabolic comorbidities, lifestyle modification should be always encouraged in these patients.

## Arterial hypertension

It is widely recognized that GC excess can increase arterial blood pressure through different mechanisms, including MC mimetic activity, alterations in peripheral and renovascular resistance, reduced nitric oxide system and vascular remodelling (20). As a direct consequence of chronic steroid therapy, hypertension is frequently observed in CAH, possibly related to supra-physiological doses of GC although not all studies are concordant (21). An increased 24h ambulatory blood pressure was confirmed in patients with CAH compared to a control group matched for age, sex and BMI (22). Despite this, few studies have assessed the association between GC dose and hypertension in CAH (17, 23–26). Regarding the type of GC used, a direct correlation was observed between blood pressure and dexamethasone treatment in a U.K. cohort of patients (27). Recently, the role of GC in arterial hypertension in adult CAH has been validated by the strong association between blood pressure and suppressed androstenedione level, reflective of excessive GC dose (19). Interestingly, the same study reported a direct correlation between MC dose and arterial blood pressure in childhood (19). Indeed, the contribution of MC replacement cannot be overlooked (4, 17, 19, 28–30) as it is associated with cardiac hypertrophy, vascular remodelling and glomerulosclerosis irrespective of blood pressure levels (31). Fludrocortisone is mainly used in SW-CAH and increases blood pressure through its direct binding with the mineralocorticoid receptor (MR) (32, 33). Periodical assessment of volume status, electrolytes and/or renin activity is suggested to avoid excessive MC replacement (2). As observed in different studies, the need for MCs varies during one’s lifetime (18, 30, 34); the physiological resistance to MCs is more pronounced during early childhood due to a lower MC sensitivity in the kidneys, and then decreases with age. Salt supplementation in infancy was associated with lower GC and MC requirement, but no differences were reported in anthropometric parameters; however, blood pressure and body weight tended to increase after the first year of life in both salt-treated and non-salt-treated children with SW form (35).

Hypertension secondary to relative MC overtreatment was probably due to a delay in fludrocortisone down-titration required during long-term follow-up (4, 9, 19, 28, 29). In a recent study Subbarayan et al. confirmed the correlation between fludrocortisone dose and diastolic blood pressure (29); interestingly, they reported an overall reduced incidence of hypertension in CAH compared to previous studies (36, 37). This change probably reflects the use of lower dosing schedules than those previously given (29). Contrastingly, some studies showed no increases in associated hypertension, probably related to both GC and MC under-treatment, as supported by the marked elevation in ACTH and plasmatic renin levels found in most of the patients included (38). A recent cross-sectional study found a direct correlation between 17-hydroxyprogesterone and diastolic blood pressure in children and adolescents with CCAH, suggesting that poorly control disease can pose the basis for cardiovascular risk as well (39).

## Vascular disease

Intima media thickness (IMT) is a widely recognized sign of arterial impairment and a predictor of future coronary artery disease and stroke (40). An increased IMT at different vascular districts (common carotid, abdominal aorta, carotid bulb, femoral arteries) was observed in adult patients with CCAH (26); however, this study did not demonstrate progression from increased IMT to atherosclerotic plaque formation. This finding was also observed in younger patients cohort (41–44), especially in hypertensive patients and in those with non-dipping profile (45); i.e. Özdemir et al. found a higher IMT and reduced aortic and carotid distensibility in patients compared to controls, suggesting the presence of early subclinical atherosclerosis (41).

This effect was also present subjects with NCCAH that exhibited higher IMT values in all main arterial districts, as observed for CCAH forms (42); moreover, it was demonstrated, in both CCAH and NCCAH, that abdominal aorta IMT, as well as common carotid artery, were positively correlated with cumulative GC doses and triglyceride serum levels and negatively with androstenedione (an indirectly confirmation of GC treatment excess).

Furthermore, some evidence indicates that IMT could be related to androgen excess (46, 47) too. Carotid IMT was associated with increased androgens (17OH-progesterone and androstenedione) in CAH adolescents and young adults (46). To further stress the importance of adequate GC therapy and androgen control, a recent study showed no increase in IMT in CAH patients, thanks to optimal androgen control in most of the cohort (7). Signs of endothelial dysfunction, measured by flow-mediated dilatation (FMD) and glyceryl trinitrate-mediated dilatation (GTN) were observed in adolescents with CAH, and the impairment was comparable to that of the obese control group (48). Furthermore, novel markers of inflammatory response such as neopterin, a catabolic product of guanosine triphosphate secreted by activated macrophages that might promote vascular dysfunction and plaque formation, were found to be increased in CCAH (49).

Despite these findings, an echocardiographic study found a preserved myocardial function in young CAH adults, although higher GC doses were associated with detrimental effects on cardiac hypertrophy, left ventricular and aortic dilation and subclinical atherosclerosis (50). An increased epicardial fat thickness was observed in children with CAH, strictly related to both BMI and waist circumference. This alteration indicates a “low-grade inflammatory status” that pave the way to the atherosclerotic process later in life (47).

## Insulin resistance and Diabetes Mellitus (DM)

Patients with CAH have an increased risk of developing insulin resistance (IR) and subsequently diabetes mellitus (DM)due to chronic GC therapy (3, 4, 17, 27, 34, 38, 51). Mechanisms behind GC-induced DM include among others, IR, increased hepatic gluconeogenesis and reduced insulin secretion (52). IR in CAH seems to be related not only to cumulative dosing, but also to GC type. A cross-sectional study on adult patients pinpointed a higher prevalence of IR in patients on long-term dexamethasone compared to those taking either prednisolone or hydrocortisone (27). Since dexamethasone is usually given at night to suppress early morning ACTH surge, its detrimental effect on glucose metabolism can be driven also by the disruption of physiological cortisol rhythmicity (53). Although IR has rarely been studied in NCCAH, its prevalence seems to be less pronounced compared to CCAH (54, 55). This may be due to higher GC doses used to suppress androgen secretion in the classic form (4).

One recent study examined a relatively small group of young patients (22, of which 13 were still on steroid therapy) using the hyperinsulinemic-euglycemic clamp, showed a higher rate of IR in both lean and obese patients with NCCAH compared to controls. Interestingly, the compensatory hyperinsulinemia was mainly sustained by a reduction of hepatic insulin clearance rather than a net increased insulin secretion (55). As previously observed for classic form, IR was associated to long-term GC therapy and especially with long-acting GC like dexamethasone (51).

Besides GC, other risk factors such as familiar history of diabetes and obesity can disrupt this delicate balance and boost the development of DM.

Androgen levels should be encountered amongst risk factors as well. To be precise, androgens have different effect on IR depending on gender; low testosterone levels promote metabolic syndrome in men (56), whilst hyperandrogenism is associated with IR, obesity and metabolic syndrome in women, as extensively demonstrated in polycystic ovarian syndrome (57). The negative impact of androgen excess in CAH was confirmed in a cohort of GC-naïve Chinese female with SV-CCAH; IR and adverse metabolic markers were significantly higher in patients compared to controls and directly related to testosterone levels (58). Two other studies supported the role of androgens in IR development in women with CAH and found that IR was more commonly associated with poor androgen control due to low GC replacement (5, 7). Therefore, supraphysiological GC treatment may not be the sole reason for impaired insulin sensitivity, especially in female patients, where androgen excess could play a relevant role.

Despite a host of risk factors, only the Swedish CAH registry found an increased prevalence of DM, especially in female patients, compared to general population (17). As for other comorbidities, it is plausible that the true extent of DM in CAH may be underestimated by the relatively young ages of CAH patients studied so far, and limited lifetime follow-up.

## Type of GC and CV risk

Unlike in childhood where hydrocortisone is the GC of choice, in adults there is little consensus on the most appropriate GC replacement regimen. Once final height is achieved, patients are frequently shifted to long-acting GCs to allow once daily dosing and consequent better treatment compliance (2). A recent meta-analysis supported a negative impact of dexamethasone on metabolic parameters compared to either prednisone or hydrocortisone (16). These detrimental effects may be at least partially due to reverse circadian dosing, with long-acting synthetic GC given at bedtime. Therefore, other GCs might be preferred over dexamethasone in the presence of metabolic comorbidities. Dual-release hydrocortisone demonstrated an improvement in glucose metabolism in patients in adrenal insufficiency compared to standard hydrocortisone replacement; however, androgen control was labile in the 6 CAH patients included and there are no data on long-term effects on CV risk (59). More promising results were provided by the use of a delayed-release hydrocortisone formulation that guaranteed a stricter adrenal androgen control with lower steroid dose (60). However, further data are required to determine whether these encouraging results can be translated into long-term cardiovascular health.

## Androgen secretion

Androgen excess is one of the hallmarks of CAH, and its control is frequently challenging. Both hypoandrogenismin males and hyperandrogenism in females can lead to adverse metabolic effects thereby increasing CV risk (23, 61, 62).

In the study of 203 patients by Arlt et al., androgens levels were poorly controlled, with most patients having either elevated or suppressed androgens, whereas only 36% presented with normal androstenedione levels (3).

Similar results were found in another study, with only half of the whole cohort having androgens level within normal range (4). New treatment approaches targeting the CRF type 1 receptor at pituitary levels (crinecerfont and tildacerfont) showed promise in reducing androgen biomarkers; ongoing studies are needed to demonstrate long-term improvement in metabolic profile (63).

## Dyslipidaemia

Dyslipidaemia does not seem to be significant in CAH patients, as shown by several studies (23, 38, 44, 48). In a cohort of 244 patients only 2% of children and 6% of adults had elevated cholesterol, but with concomitant increase in HDL (4). Very few studies showed alterations in blood lipid profile; Arlt et al. found high cholesterol levels in 46% of adult patients (3) whereas a recent American study of children and young adults showed an increased incidence of low HDL (57.9%) and high triglycerides (42.1%), although this cohort was biased by a higher rate of obesity compared to other European series (19). The same study suggested a detrimental role of GCs on lipid profile in children, with higher total cholesterol and LDL in those with tighter disease control. Similarly, androstenedione suppression and excessive MC therapy were risk factors for low HDL and high LDL, respectively, in adult patients (19). However, as demonstrated by Zhang et al. on 30 untreated females with SV form, higher TG and lower HDL-cholesterol were associated with androgens excess (58). By the same token, dyslipidaemia is a common finding even in NCCAH (3). Surprisingly, when compared to a controls matched for sex, age and BMI, patients with CAH tended to have higher HDL-cholesterol and adiponectin (22).

It should be mentioned that most available studies involved young patients, thus the true estimate of lipid impairment might be under-representative.

## The role of GC receptors polymorphism in CAH patients

Apart from cumulative GC dose, the effect of systemic therapy can be modulated at receptor level; polymorphisms of the GC receptor gene (*NR3C1*) can be either associated with adverse or beneficial metabolic and cardiovascular profiles in general population. The ER22/23EK haplotype is associated with reduced GC sensitivity, thus to a more favorable metabolic profile, whilst the N363S and *Bcl*I restriction fragment length polymorphism (RFLP) cause GC hypersensitivity (64). As a result, patients harboring the N363S mutation, have a higher incidence of type 2 DM, obesity and CV diseases (65, 66). Similarly, individuals with the *Bcl*l mutated population has higher prevalence of obesity and hypertension (67). GC polymorphisms have been studied in CAH patients as well. Heterozygotes for the *Bcl*I haplotype present with higher BMI, waist circumference and higher systolic blood pressure compared to wild-type subjects (68), even though the frequency of this polymorphism was less common in patients with CAH than in general population (69).

The A3669G-polymorphism was instead inconsistently associated with adverse lipid profile in paediatric CAH patients (68, 70). *NR3C1* genotyping might play an important role in predicting individual response to GC therapy and identify patients at high risk for GC-related complications.

## Cardiovascular events

Most of the studies on metabolic risk include children or young adults, thus longitudinal studies on subject aged greater than 30 years are scant. A Swedish cohort of 588 patients with CAH of all ages showed an increased risk of CV disease (OR 2.7) compared to reference population; however, no significant differences for cardiac arrest, heart failure and atrial fibrillation were observed. An increase incidence of stroke was reported only for female with NCCAH. Instead, acute coronary syndrome was significantly increased in males with SW-CAH, especially in those with null genotype (17). Also increased thromboembolic events, mostly in female patients, was reported, potentially related to obesity and GC-induced hypercoagulability. However, the contribution of GC therapy to these events was not assessed (17). The same authors found that CV events were the second leading cause of mortality, although half of these patients had a concomitant infection that might have resulted in an overestimation of the CV-related death due to a concomitant adrenal crisis (71).

Interestingly, in the UK cohort only a minority of adults affected by CAH are regularly follow-up by an endocrinologist; this lack of disease-monitoring might negatively impact on patients’ well-being and contribute to their mortality rate (3).

## Cardiovascular mortality

To date, few studies have assessed disease mortality in adults. Fahlhammar et al. found a higher mortality rates in patients with CAH, the main causes of death were adrenal crisis (42%), CV disease (32%), cancer (16%) and suicide (10%) (71). However, it should be mentioned that false inflation of mortality rates may have been due to increased perinatal mortality prior to introduction of neonatal screening. After excluding of patients who died within the first year of life in whom the correct diagnosis was probably missed, the mortality rate remained higher only for SW-CCAH form and in affected females. A U.K. study found a greater all cause-mortality risk in CAH patients; the mean age at death was 50 years but the underlying causes were not reported (72). Overall, there has been an increase in survival over time reflecting the improvements in diagnostic criteria and GC treatment; the mean age of death in the Swedish CAH population has increased from 21 years during the 1980s to 57 years in 2010 (71).

These data were also confirmed in a recent German study which found an increased mortality in the period between 1973 and 2004 (even after introduction of general screening), mainly related to inadequate GC stress-dose administration, but no further deaths thereafter, supporting the importance of patient education in preventing adrenal crisis (73).

## Cardiovascular health in uncommon forms of CAH

**Figure 2:**
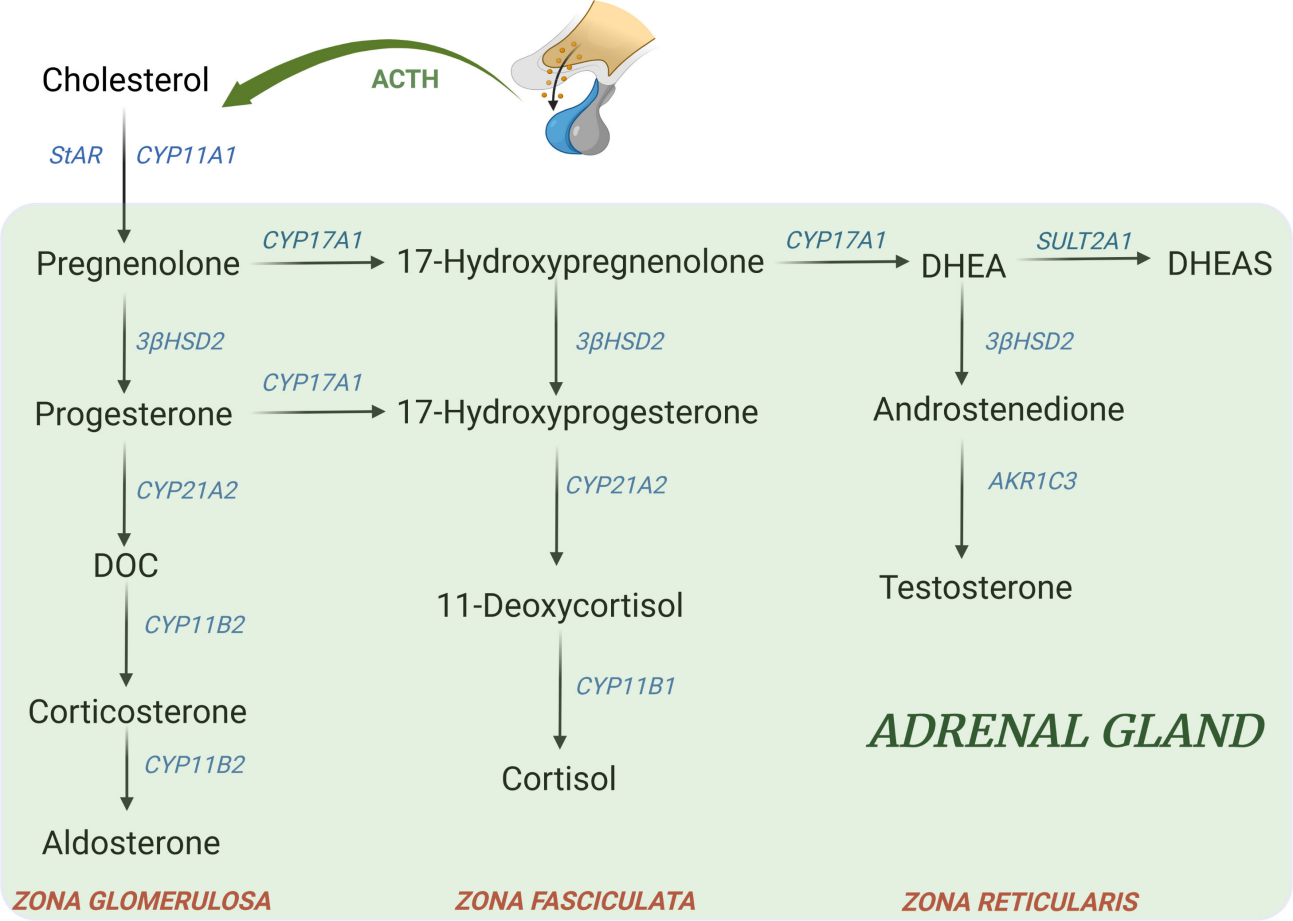
Major adrenal steroid synthesis pathways. Uncommon CAH forms include: - 11β-hydroxylase deficiency (11-OHD): block at CYP11B1 → ↓ cortisol; ↑ DOC, ↑ 17-hydroxyprogesterone, androgens, ↑ 11-deoxycortisol - 17α-hydroxylase deficiency (17-OHD): block at CYP17A1 → ↓ 17-hydroxyprogeserone, ↓ androgens; ↑ DOC, ↑ corticosterone - 3β-hydroxysteroid dehydrogenase type II deficiency (3β-HSD): block at 3β-HSD → ↓ aldosterone, ↓ androgens in male; ↑ 17hydroxypregnenolone, ↑ 17-hydroxyprogesterone, ↑ DHEA - Lipoid form (LCAH)→ block at StAR level → ↓ all steroids - P450 oxidoreductase deficiency (PORD): multiple partial blocks of CYP17A1 and CYP21A2→ ↓ androgens; ↑ progesterone, ↑ 17-hydroxyprogesterone, ↑ corticosterone (74). DOC, 11-deoxycorticosterone; DHEA, dehydroepiandrosterone; DHEAS, dehydroepiandrosterone sulfate.

Other genetic forms account together for the remaining 5% of all cases of CAH. According to the defective enzyme, the resulting changes include a deficiency of steroids downstream from the block and an accumulation of precursors upstream that can be shunted to other pathways, **Figure 2** (74).

Patients with 11β-hydroxylase deficiency (11-OHD) have deficient aldosterone and cortisol production but elevated androgens and MC precursors (75). Arterial hypertension is found in up to two thirds of patients with classic 11-OHD (76–78) and it could appear in childhood or even only later in life (79, 80); non-classic forms are often normotensive at diagnosis (81). Usually hypertension is mild, but severe cases that required bilateral adrenalectomy, have also been described (82–85). Moreover, malignant hypertension can lead to left ventricular hypertrophy, retinopathy, ischemic heart disease, nephropathy, cerebrovascular events and also death (77, 78, 84–86). These complications were mainly reported in poorly controlled patients as indirectly documented by the severity of virilization (78). Other comorbidities have been rarely described in 11-OHD; obesity is less frequent in 11-OHD compared to 21-OHD (78, 81, 87–89), as observed in a *Cyp11b1* null mouse model, which reflects the absence of direct GC effect on adipose tissue (90). Insulin resistance was found in 10.7% of the population analyzed (78) potentially related to MC excess and hypokalemia (90), but overt diabetes mellitus has been rarely reported (91).

Although there are no studies that directly assessed mortality in 11-OHD, these patients seem to have an increased risk of CV events but fewer adrenal crisis compared to patients with CCAH (77, 92, 93); however, they can develop adrenal insufficiency if GC therapy is withdrawn, because 11-deoxycorticosterone (DOC) alone is not sufficient to prevent adrenal crisis during stressful events (94).

The 17α-hydroxylase deficiency (17-OHD) is the second most common CAH in Brazil; besides MC excess, patients display androgen and estrogen deficiency but they do not manifest adrenal insufficiency thanks to corticosterone excess (74). In a Brazilian cohort of 24 patients with 17-OHD two hypertensive young patients of 34 and 27 years had a history of stroke (95). However, a possible contribution of lifestyle or other genetic factors was advocated as a potential confounding factor (95, 96).

Studies on patients with 3β-hydroxysteroid dehydrogenase type II deficiency (3β-HSD) included neonatal or children, thus there are no data on CV risk in adolescents/adults (97).

The lipoid form (LCAH) is the second most common type of CAH in Japan and Korea (98). The largest study including 57 patients, 43 with classical form, (aged 0.0–47.5 years) did not report CV events (99). To date, there are no longitudinal studies on CV risk in LCAH patients with classical form (100). Similarly, the prevalence of CV risk factors in the milder non-classic form of LCAH (NCLAH) are lacking (100). There is just one case report of an overweight 26 years old female patient with NCLAH who had concomitant borderline blood pressure but normal HbA1c and lipid profile (101).

## Conclusions

Diagnostic and treatment developments over the years have normalized life-expectancy in patients with CAH. Despite this, series on aging CAH patients are still limited in literature. To date, most available studies pointed to an increase cardio-metabolic risk in CAH patients. Features of metabolic syndrome can appear early in childhood and should be rapidly addressed to reduce future CV events. GC overtreatment seems to have a pivotal role in promoting weight gain, which represents the main independent risk factor for the development of metabolic syndrome and directly contributes to hypertension, IR and hypercoagulability. Long-acting GCs may be associated with adverse metabolic profile, but current data are not sufficient to draw conclusions on the best GC schedule for adult patients. Similarly, excessive MC replacement, particularly if not properly down-titrated in adulthood, can contribute to the increased CV risk. On the other hand, androgen excess can be equally harmful and negatively impact on several metabolic aspects, as observed in NCCAH. As a general rule, patients with CAH should receive the lowest possible GC dose to prevent adrenal insufficiency and signs and symptoms of hyperandrogenism. A periodic screening for arterial hypertension and metabolic comorbidities should be performed since early infancy to avoid future CV events. Similar conclusions can be drawn also for rarest forms of CAH, although the CV risk profile of these patients is less characterized due to the low number of studies available. Ongoing and future studies will clarify whether new compounds might promote reduced GC overexposure and guarantee a more stable disease control resulting in a more favorable metabolic profile. Overall, available data pointed to an increased mortality in CAH patients, probably biased by older studies when neonatal screening was not available and GC replacement not appropriate. Conversely, CV morbidity and mortality might be significantly underestimated, since most studies analyze adolescents or young adults, while CV events usually occur later in life. Future perspective can include GR polymorphisms genotyping to identify patients at higher CV risk and allow a personalized GC treatment to avoid long-term adverse consequences.

## Author contributions

MB and CS contributed to conception of the study. PM and ML performed bibliographic research. MB, PM, and ML were involved in drafting the manuscript. PM and FC were in charge of figures. MB, FC, and CS were involved in manuscript revision. All authors contributed to the article and approved the submitted version.

## Conflict of interest

The authors declare that the research was conducted in the absence of any commercial or financial relationships that could be construed as a potential conflict of interest.

## Publisher’s note

All claims expressed in this article are solely those of the authors and do not necessarily represent those of their affiliated organizations, or those of the publisher, the editors and the reviewers. Any product that may be evaluated in this article, or claim that may be made by its manufacturer, is not guaranteed or endorsed by the publisher.
